# Inadvertent, intraoperative, non- to minimally displaced periprosthetic humeral shaft fractures in RTSA do not affect the clinical and radiographic short-term outcome

**DOI:** 10.1007/s00402-021-03930-z

**Published:** 2021-06-05

**Authors:** Anita Hasler, Philipp Kriechling, Caroline Passaplan, Karl Wieser

**Affiliations:** Department of Orthopaedics, University Hospital Balgrist, Zurich, Switzerland

**Keywords:** Intraoperative complications, Reverse total shoulder arthroplasty, Periprosthetic humeral fracture

## Abstract

**Introduction:**

Little information is available on the clinical and radiographic outcome of intraoperative, non- to minimally displaced humeral fractures that occur during implantation of a stemmed, reverse shoulder prosthesis but are only recognized on routine postoperative radiographs. The goal of this study is to report the clinical and radiographic outcome for this rarely reported fracture type.

**Materials and methods:**

39 conservatively treated non- to minimally displaced intraoperative periprosthetic humeral fractures after stemmed RTSA were detected from our radiographic database between 1.1.2006 and 31.1.2018. Exclusion criteria were lack of patient consent, preoperative humeral fracture, and revision arthroplasties. Clinical (absolute and relative Constant score, the Subjective Shoulder Value) and radiographic (conventional radiographs) assessment was performed preoperatively, at 6 weeks (only radiographically) and at latest follow-up with a minimum follow-up of 2 years.

**Results:**

35 patient’s with a mean age of 72 years (range 32–88, SD ± 11 years) and a mean follow-up of 53 months (range 24–124, SD ± 31) were included in the study. At latest follow-up, all clinical outcome parameters except external rotation improved significantly. A complication rate of 17% (*n*:6) was recorded. At 6 weeks after the index surgery, none of the radiographs showed a fracture displacement or a sintering of the stem. At latest follow-up, all fractures were healed and no stem loosening was observed in any of the shoulders.

**Conclusions:**

Non- to minimally displaced intraoperative periprosthetic humeral fractures in RTSA have an incidence of about 5% in this series of mainly uncemented press-fit stems. They generally heal without any further treatment and are not associated with stem loosening or compromise the clinical outcome after primary RTSA. Except slight restriction in the postoperative rehabilitation protocol, no further attention or action is needed.

## Introduction

Reported complications of reverse total shoulder arthroplasty (RTSA) include in decreasing frequency prosthetic instability, infection, humeral problems (loosening and fracture), glenoid loosening, acromial/scapular spine fractures, neurologic complications and others [1–4].

Periprosthetic humeral fractures can occur intraoperatively or postoperatively, and are considered to be challenging and associated with a high complication rate after revision surgery [5]. The incidence of intraoperative periprosthetic humeral fractures is approximately 1.5–16% [6–10] with one study of Athwal et al. dealt exclusively with isolated intraoperative fractures [11].

Risk factors for intraoperative humeral fractures are female sex [odds ratio (OR), 4.19] [8], posttraumatic arthritis (OR 2.55) [8] and press-fit humeral components [relative risk (RR) 2.9] compared with cemented components [11]. Other risk factors are revision surgery [11, 12] and history of instability. In primary shoulder arthroplasty, mechanisms of fractures are endosteal notching from reaming [13], excessive humeral external rotation during exposure, and cortical breaching while reaming or broaching for implant insertion [14, 15]. Treatment recommendations for periprosthetic humeral fractures consider the fracture type, location and implant stability. The recommendations vary from non-operative management to open reduction and internal fixation (ORIF) to revision arthroplasty [16]. There are various classification systems for periprosthetic shoulder fractures, the latest of which also provides a structured treatment algorithm, is published and validated by Kirchhoff et al. [17, 18]. All their reported cases are postoperative fractures and an algorithm for intraoperative fractures is not available.

To our best knowledge, there is little information available regarding the rehabilitation recommendation or outcome of those non- to minimally displaced intraoperative periprosthetic humeral fractures after RTSA, that are incidentally detected on the first postoperative radiograph. During the surgery, this fracture type usually remains undiscovered, if good press fit or hold can be achieved with the final implant and no obvious fracture line is seen. The goal of this study was to present the clinical and radiographic outcome of such non- or minimally displaced periprosthetic humeral shaft fractures after primary RTSA with a minimum follow-up of 2 years.

## Materials and methods

**Fig. 1:**
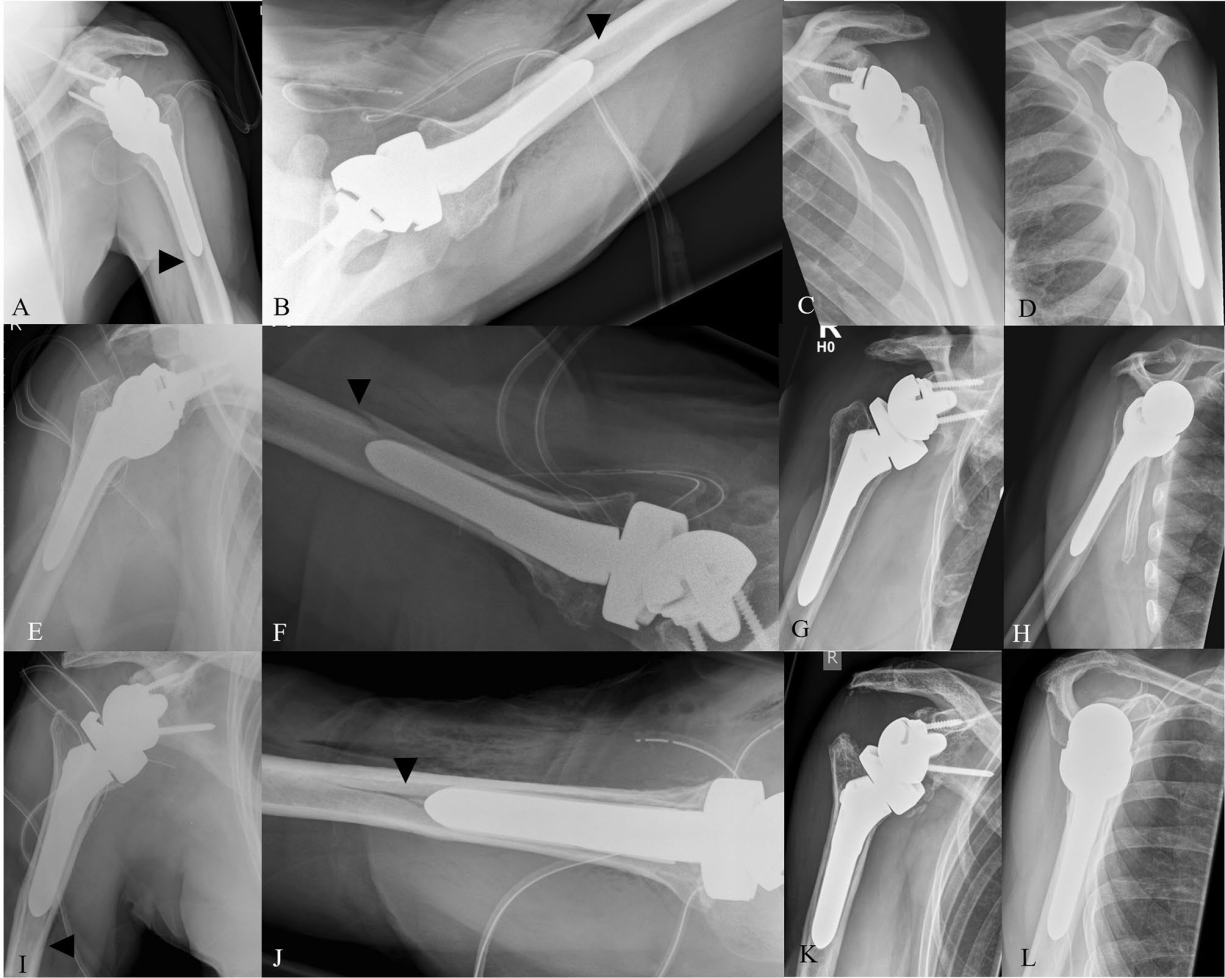
These radiographs show various fracture types with there follow-up.** A**/**B** 65-year-old man with irreparable rotator cuff tear. On the initial radiograph after the implantation of a RTSA, a non-displaced, oblique humeral shaft fracture is visible at the distal end of the stem on the anteroposterior as well as on the axial view.** C**/**D** 5-year follow-up of the case. No radiolucency of the shaft is visible.** E**/**F** Radiographs of a 72-year-old woman treated with a RTSA for irreparable rotator cuff lesion. The postoperative radiographs show a slightly displaced, spiral humeral shaft fracture. The fracture is only visible on the axial view.** G**/**H** 2-year follow-up. No radiograph complication can be detected at last follow-up.** I**/**J** Radiographs of a 79-year-old woman, treated with a RTSA because of irreparable rotator cuff tear. Also, in this case, the hypodense fracture line is only visible on the axial view and seems to be bifurcated.** K**/**L** 5-year follow-up of the case. No radiolucency of the shaft is visible.

The responsible review board approved this study (KEK-ZH-Nr.2018-01494). Between January 2006 and January 2018, 782 primary RTSAs were performed in our institution. Inclusion criterion for this investigation was the occurrence of a periprosthetic humeral shaft fracture after RTSA, which was detected incidentally on the first postoperative radiograph (anteroposterior radiographs and axial views, taken in the theater after wound closure). Therefore, immediate postoperative X-rays of all patient’s after primary RTSA were retrospectively screened for the occurrence of a non- or minimally (defined as less than 3 mm) displaced intraoperative periprosthetic fracture line. We could detect 39 patient’s, giving a periprosthetic incidence of 5%. (Fig. 1) For the fracture pattern, we had a consensus agreement by two independent reviewers

Exclusion criteria were lack of patient consent, conservatively or operatively treated preoperative humeral fractures and revision arthroplasties. Also, all other fracture patterns which occurred during the surgery and needed strategy change during the initial surgery were excluded (for example, the use of a revision stem or cerclage because of intraoperative fracture of the stem, fracture of the tuberosities, in total: *n*:80, 10%).

All these fractures were unnoticed during surgery and, therefore, no special treatment was performed intraoperatively. No special bracing was performed in all of these cases but the postoperative rehabilitation protocol was adjusted to sling protection without active postoperative joint mobilization in four of the cases. These modifications were individual precaution by the surgeons and not related to fracture type or patient’s characteristics.

All other shoulders were treated with a standard postoperative rehabilitation protocol, with passive and active-assisted joint mobilization and a sling for comfort use. In case of subscapularis repair, the external rotation was limited to 0° for the first 6 weeks. One patient was treated with a brace in neutral position because of an additionally performed l'Episcopo transfer.

Four patient’s did not sign an informed consent and declined to be part of the study. Finally, 35 shoulders in 35 patient’s were available for further analysis; 24 patient’s (69%) were treated for irreparable rotator cuff tear with mild or severe osteoarthritis and 11 patient’s (31%) for primary or secondary osteoarthritis. Demographic parameters (age, gender, American Society of Anesthesiologists (ASA) score, BMI, comorbidities) and surgery-related characteristics (use of cement for the humeral stem) were evaluated.

35 patient’s (11 men and 24 women; mean age 72 years (32–88, SD ± 11) were examined with a mean follow-up of 53 months (24–124, ± 31). The dominant shoulder was involved in 23 cases (66%) cases (left: 12 (34%), right: 23 (66%)).

The ASA score was I in 1 patient (3%) and II in the majority of 26 patient’s (74%). 8 patient’s (23%) had an ASA score of III. The mean BMI was 27 kg/m^2^ (18–39, ± 5).

Regarding comorbidities, 2 patient’s had diabetes mellitus and another two patient’s were under long-term steroid use. One patient had Parkinson disease. Seven patient’s had osteoporosis with medical treatment.

### Clinical and radiographic assessment

Clinical examination was performed preoperatively and on regular follow-up visits (2y, 5y, 7.5y, 10y, and afterwards every 5 years until revision or patient's death).

Clinical examination included assessment of the absolute Constant score (aCS) [19], the relative Constant score (rCS) [20] and the Subjective Shoulder Value (SSV) [21]. Pain level was measured with a visual analog scale (Constant pain scale) on which 15 indicates no pain and 0 indicates intolerable pain. Active range of motion was measured using a handheld goniometer and abduction strength was measured with a validated electronic dynamometer (Isobex; Cursor).

Preoperatively and postoperatively, standardized true anteroposterior radiographs and axial views were obtained for all patient’s at every follow-up, but Neer views were performed only preoperatively, at 6 weeks and latest follow-up. Computer tomography (CT) was obtained preoperatively in all cases. All images were reviewed by a fellowship-trained shoulder surgeon (AH). Preoperatively acromiohumeral distance (ACHD) and bone quality (deltoid tuberosity index [22]) were evaluated as well as the grade of rotator cuff arthropathy according to the Hamada classification [23]. On preoperative CT scans, glenoid dysplasia was graded according to Walch [24]. On the initial postoperative radiographs, the periprosthetic fractures were analyzed for fracture type (transverse, oblique, spiral, or comminuted) and maximal dislocation in mm was measured. Additional fractures on the medial calcar, lesser and greater tuberosities were documented. Outcome measurements on the radiographs at 6 weeks postoperatively were fracture displacement, stem sintering/loosening and whether the fracture was still visible. At latest follow-up, fracture union and humeral stem lucency/loosening were recorded. Fracture union was defined as the presence of bridging bone or the disappearance of the fracture line on two orthogonal views. Additionally, glenoid loosening was assessed.

### Surgical technique

All shoulders were exposed through a deltopectoral approach. In all cases, the Anatomical Shoulder™ Inverse/Reverse prosthesis (Zimmer-Biomet^®^) was implanted. The humerus was prepared first and the trial implant was left within the humeral canal during the implantation of the glenoid component. In case of sufficient bone quality (intraoperatively judged by the responsible surgeon), a press-fit humeral stem fixation was performed if possible (*n* = 27, 77%). Due to missing press fit, the humeral component was cemented in place with gentamicin-impregnated bone cement (Palacos; Heraeus Kulzer) in 8 shoulders (23%). If possible, the subscapularis tendon was reattached to the lesser tuberosity at the end of the procedure (*n* = 28). In all cases, the surgeon did not realize the fracture at the distal end of the humerus during the surgery with a stable and good fit of the stem. Therefore, no special intervention like cerclage wiring, plate fixation or even revision to a longer stem was performed.

### Statistical analysis

Patient’s data were collected in REDCap^®^ Electronic Data Capture system version 8.6.1 (Vanderbilt University, 1211 Medical Center, TN 37232, USA) anonymously.

For statistical analyses, IBM SPSS Statistics software for Windows version 24.0 (IBM Corp., NY, USA) was used. Values were expressed as mean, range and ± standard deviation. Data were tested for normal distribution using the Kolmogorov–Smirnov test. We utilized paired and unpaired t-test for parametric data, Wilcoxon or Mann–Whitney U for non-parametric data and Fisher's exact test for categorical variables. A *p*-value of less than 0.05 was considered significant.

## Results

### Preoperative radiographic findings

In 24 cases (69%), the deltoid tuberosity index [22] was lower than 1.4, indicating a low local bone material density of the proximal humerus. In the majority of the cases, the glenoid type was A1 or A2, with only 3 cases of each a B2 or C type glenoid according to Walch [24]. Complete morphological characteristics are depicted in Table 1.

**Table 1:** Preoperative radiographic findings

Entire series (*n* = 35)	Mean (range, ± SD) or exact number (%)
ACHD (mm)	6 (1–17, ±4.1).
Deltoid tuberosity index	1.4 (1.1–1.7, ±0.1)
Hamada classification	
Grade I	12 (34%)
Grade II	21 (60%)
Grade III	2 (6%)
Glenoid type according to Walch	
A1	22 (63%)
A2	7 (20%)
B2	3 (9%)
C	3 (9%)

### Clinical outcome

Full clinical data were available for all patient preoperatively and for 33 patient’s postoperatively. At latest follow-up, aCS, rCS, SVV, and pain level had improved significantly from preoperatively (*p* < 0.001). The mean aCS increased to 67 points (31–86, ± 14), the mean rCS to 82% (43–104,± 15) and the mean SSV to 81% (30–100, ± 19), respectively. The pain score improved to a mean of 14 points (3–15, ± 3). Active ranges of flexion, abduction and internal rotation were significantly improved at latest follow-up (*p* = 0.007, < 0.001 and 0.021). External rotation did not significantly change after the implantation of the RTSA (*p* = 0.409). For complete pre- and postoperative data, see Table 2.

**Table 2:** Clinical outcome

Entire series (*n* *=* 35)	Preoperative (*n*:35)	Last follow-up (*n*:33)	*P* value
SVV (%)	36 (0–80, ± 19)	81 (30–100, ± 19)	** < 0.001**
aCS (points)	40 (6–68, ± 16)	67 (31–86, ± 14)	** < 0.001**
rCS (%)	50 (8–85, ± 19)	82 (43–104, ± 15)	** < 0.001**
Pain score (points)	7 (0–15, ± 4)	14 (3–15, ± 3)	** < 0.001**
Abduction force (kg)^a^	2 (0–16, ± 3)	3 (0–7, ± 2)	**0.005**
Range of motion			
Flexion (°)	98 (20–160, ± 43)	121 (50–165, ± 22)	** < 0.001**
Abduction (°)	85 (30–160, ± 37)	128 (40–175, ± 31)	** < 0.001**
Internal rotation (points) ^b^	5 (0–10, ± 2)	6 (2–10, ± 2)	**0.021**
External rotation (°)	25 (-25–90, ± 29)	28 (-20–75, ± 21)	0.409

Values reported in mean (range) and ± standard deviation, significant *p*-values are bold

^a^Mean value of 3 trials

^b^Internal rotation: 0–10 points, End of the thumb defined the score, 0 points: lateral thigh, 2 points: buttock, 4 points: lumbosacral junction, 6 points: L3, 8 points T12, 10 points: T7

### Complication*s*

Despite the postoperatively detected fractures, there were no other documented intraoperative complications. After surgery, 6 (17%) complications were recorded in 5 patient’s. There was one acromion fracture (seen at 6-month follow-up, treated conservatively) and one with an atraumatic, minimally displaced greater tuberosity fracture seen 6 weeks after the surgery, without further treatment. One patient had a hematoma and prolonged wound secretion for 13 days (Ultrasound proven, no intervention needed). Two plexus injuries occurred after surgery with unknown injury mechanism, but possible due to traction during surgery. In one patient, the lesion was transient and she fully recovered after 6 months. The second patient had a complete plexus lesion after the index surgery without known origin. The symptoms improved clinically and on electromyography after 10 months postoperatively. However, due to ongoing painful shoulder stiffness, an arthroscopic capsulotomy was performed 14 months postoperatively. At latest follow-up (48 months), she had a SSV of only 30% with ongoing pain but with acceptable range of motion (Flexion 120°, abduction 110°, external rotation 40°). She died unrelated to her shoulder 3 years after surgery.

### Radiographic outcome

Complete radiographic follow-up was available for all 35 patient’s direct postoperatively, at 6 weeks and at no less than 2 years. At the initial postoperative radiograph, the fracture was visible as a small crack (hypodense line in the radiograph) in extension of the stem (sometimes, there was a forked-line fracture) but without displacement (oblique fracture type) in 33 of the cases (94%). In two patient’s (6%), the fracture line was extending spiral shaped in the other cortex with minimal displacement (spiral fracture type) (See Fig. 1). The displacement was 1.5 and 2.2 mm, respectively. No transverse or comminuted fracture was detected. In 9 cases, an additional fracture line was seen on the level of the medial calcar. There was neither a greater nor lesser tuberosity fracture on the initial postoperative radiographs. At 6 weeks after the index surgery, none of the radiographs showed a fracture displacement or sintering of the stem. In 49% of the cases (*n*:17), the fracture line was still visible at least on one radiograph. At latest follow-up, all fractures were healed (Fig. 1).

No stem or glenoid loosening was observed in any of the shoulders. In one patient, the uncemented stem showed a radiolucent line around the medial side of the stem. Compared with the immediate postoperative radiographs, the stem was slightly more in varus without any effect on the clinical outcome of the patient (SVV 80%, rCS 69%).

## Discussion

The most important finding of this study is that incidentally detected, non- to minimally displaced periprosthetic humeral fractures heal without further clinical or radiographic complications at a minimum follow-up of 2 years.

Although there are many publications on complications of RTSA including periprosthetic humeral fractures [1–5], this fracture pattern has not yet been described in detail. The incidence of intraoperative periprosthetic humeral fractures is described with a wide range from 1.5 to 16% [6–10]. Without a systematic search in a radiographic database, these fractures might frequently not be detected and are, therefore, potentially underreported. In our database of 782 primary RTSAs, we identified 35 incidental non- to minimally displaced periprosthetic humeral fractures (5%). Because this complication was never described in the operative note, we were not able to fully reconstruct the fracture mechanism. We hypothesize that these fractures either happened with the broach or during implantation of the definitive stem. It is our current practice to prefer cementless stem fixation (adequate bone quality) but in this study, 8 of the 35 stems were cemented (23%). Thus, these fractures do also occur in cemented prostheses, although they are more common in the uncemented than in the cemented RTSA (6.02% vs 1.2%) [10, 25].

To minimize this risk, the size of the stem is planned preoperatively on an anterior–posterior and axillary lateral radiograph. This two-dimensional planning could, however, be misleading—with oversizing the stem—as a cadaver study of Lee et al. showed that the average medial–lateral endosteal diameter (15.6 ± 2.3 mm) was significantly greater than the anterior–posterior diameter (12.5 ± 1.9 mm) at 13 cm distal to the tuberosity [13]. Further, they could show that intramedullary reaming removes cortical bone asymmetrically and may potentially increase the risk of periprosthetic fractures.

The fracture was visible in 33 cases as a small crack distal to the stem but without displacement. In two cases, the fracture line was extending spiral shaped in the opposite cortex with minimal displacement. At final follow-up, all fractures were healed and no stem loosening could be detected. Some fractures were difficult to visualize on the radiographs at 6 weeks postoperative and, therefore, an exact time to bony healing could not be calculated. Athwal et al. could also show a 100% healing rate in their study of intraoperative humeral fracture and theorized that the high union rate was favored because of low-energy trauma and, therefore, unlikely disruption of the periosteal and intramedullary blood supplies [11].

The overall clinical outcomes in this series of RTSA are comparable with averages from historical series of RTSA in previously published reports from our institution [27, 28]. The study group showed a significant pain relief and improvement in all functional outcome parameters, except external rotation. This is not unexpected since these incidentally reported periprosthetic humeral fractures did not show any radiological complications.

The main limitation is the retrospective nature of the study. At final follow-up, radiolucency and loosening were only assessed on radiographs and a more sensitive diagnostic tool as computed tomography, was not available. Due to the study design, a proper risk factor analysis was not possible. However, some findings might be highlighted. The mean age in the study group was 72 years with 67% women. Only two patient’s had long-term steroid use and 7 patient’s (20%) had osteoporosis. However, 69% of the patient’s had a deltoid tuberosity index that was lower than 1.4, indicated low local bone material density of the proximal humerus (mean 1.4, range 1.1–1.7, ± 0.1) and it is, therefore, likely that some of them had an undiagnosed osteoporosis.

Despite these limitations, the findings of this study add a valuable aspect to scientific knowledge in shoulder surgery. In contrast to ordinary reported periprosthetic humeral fractures around reversed shoulder stems, which are considered to be serious complications that worsen the clinical outcome of the patient [26], the above-mentioned fracture type might be skillfully neglected.

## Conclusion

Non- to minimally displaced intraoperative periprosthetic humeral fractures in RTSA have an incidence of about 5% in this series of mainly uncemented press-fit stems. They generally heal without any further treatment and are not associated with stem loosening or compromise the clinical outcome after primary RTSA. Except slight restriction in the postoperative rehabilitation protocol, no further attention or action is needed.

## References

[1] Boileau P (2016). Complications and revision of reverse total shoulder arthroplasty. Orthop Traumatol Surg Res.

[2] Zumstein MA, Pinedo M, Old J, Boileau P (2011). Problems, complications, reoperations, and revisions in reverse total shoulder arthroplasty: a systematic review. J Shoulder Elbow Surg.

[3] Ascione F, Domos P, Guarrella V, Chelli M, Boileau P, Walch G (2018). Long-term humeral complications after Grammont-style reverse shoulder arthroplasty. J Shoulder Elbow Surg.

[4] Wierks C, Skolasky RL, Ji JH, McFarland EG (2009). Reverse total shoulder replacement: intraoperative and early postoperative complications. Clin Orthop Relat Res.

[5] Andersen JR, Williams CD, Cain R, Mighell M, Frankle M (2013). Surgically treated humeral shaft fractures following shoulder arthroplasty. J Bone Joint Surg Am.

[6] Boyd AD, Thornhill TS, Barnes CL (1992). Fractures adjacent to humeral prostheses. J Bone Joint Surg Am.

[7] Worland RL, Kim DY, Arredondo J (1999). Periprosthetic humeral fractures: management and classification. J Shoulder Elbow Surg.

[8] Singh JA, Sperling J, Schleck C, Harmsen W, Cofield R (2012). Periprosthetic fractures associated with primary total shoulder arthroplasty and primary humeral head replacement: a thirty-three-year study. J Bone Joint Surg Am.

[9] Atoun E, Van Tongel A, Hous N, Narvani A, Relwani J, Abraham R, Levy O (2014). Reverse shoulder arthroplasty with a short metaphyseal humeral stem. Int Orthop.

[10] Garcia-Fernandez C, Lopiz-Morales Y, Rodriguez A, Lopez-Duran L, Martinez FM (2015). Periprosthetic humeral fractures associated with reverse total shoulder arthroplasty: incidence and management. Int Orthop.

[11] Athwal GS, Sperling JW, Rispoli DM, Cofield RH (2009). Periprosthetic humeral fractures during shoulder arthroplasty. J Bone Joint Surg Am.

[12] Wagner ER, Houdek MT, Elhassan BT, Sanchez-Sotelo J, Cofield RH, Sperling JW (2015). What are risk factors for intraoperative humerus fractures during revision reverse shoulder arthroplasty and do they influence outcomes?. Clin Orthop Relat R.

[13] Lee M, Chebli C, Mounce D, Bertelsen A, Richardson M, Matsen F (2008). Intramedullary reaming for press-fit fixation of a humeral component removes cortical bone asymmetrically. J Shoulder Elbow Surg.

[14] Campbell JT, Moore RS, Iannotti JP, Norris TR, Williams GR (1998). Periprosthetic humeral fractures: mechanisms of fracture and treatment options. J Shoulder Elb Surg.

[15] Williams GR, Iannotti JP (2002). Management of periprosthetic fractures — the shoulder. J Arthroplasty.

[16] Fram B, Elder A, Namdari S (2019). Periprosthetic humeral fractures in shoulder arthroplasty. JBJS Rev.

[17] Kirchhoff C, Beirer M, Brunner U (2016). Periprosthetic humeral fractures: strategies and techniques of revision arthroplasty. Unfallchirurg.

[18] Kirchhoff C, Beirer M, Brunner U, Buchholz A, Biberthaler P, Cronlein M (2018). Validation of a new classification for periprosthetic shoulder fractures. Int Orthop.

[19] Constant CR, Murley AH (1987). A clinical method of functional assessment of the shoulder. Clin Orthop Relat Res.

[20] Constant CR, Gerber C, Emery RJ, Sojbjerg JO, Gohlke F, Boileau P (2008). A review of the constant score: modifications and guidelines for its use. J Shoulder Elbow Surg.

[21] Gilbart MK, Gerber C (2007). Comparison of the subjective shoulder value and the constant score. J Shoulder Elbow Surg.

[22] Spross C, Kaestle N, Benninger E, Fornaro J, Erhardt J, Zdravkovic V, Jost B (2015). Deltoid tuberosity index: a simple radiographic tool to assess local bone quality in proximal humerus fractures. Clin Orthop Relat Res.

[23] Hamada K, Fukuda H, Mikasa M, Kobayashi Y (1990). Roentgenographic findings in massive rotator cuff tears. A long-term observation. Clin Orthop Relat Res.

[24] Walch G, Badet R, Boulahia A, Khoury A (1999). Morphologic study of the glenoid in primary glenohumeral osteoarthritis. J Arthroplasty.

[25] King JJ, Farmer KW, Struk AM, Wright TW (2015). Uncemented versus cemented humeral stem fixation in reverse shoulder arthroplasty. Int Orthop.

[26] Wolf H, Pajenda G, Sarahrudi K (2012). Analysis of factors predicting success and failure of treatment after type B periprosthetic humeral fractures: a case series study. Eur J Trauma Emerg Surg.

[27] Ek ET, Neukom L, Catanzaro S, Gerber C (2013). Reverse total shoulder arthroplasty for massive irreparable rotator cuff tears in patient’s younger than 65 years old: results after five to fifteen years. J Shoulder Elbow Surg.

[28] Hasler A, Fornaciari P, Jungwirth-Weinberger A, Jentzsch T, Wieser K, Gerber C (2019). Reverse shoulder arthroplasty in the treatment of glenohumeral instability. J Shoulder Elbow Surg.

